# Variations in inter‐specific and sex‐related niche partitioning in pelagic boobies during their annual cycle

**DOI:** 10.1002/ece3.11255

**Published:** 2024-04-15

**Authors:** Miriam Lerma, Nina Dehnhard, José Alfredo Castillo‐Guerrero, Salvador Hernández‐Vázquez, Christian C. Voigt, Stefan Garthe

**Affiliations:** ^1^ Research and Technology Center (FTZ) University of Kiel Büsum Germany; ^2^ Norwegian Institute for Nature Research (NINA) Trondheim Norway; ^3^ Departamento de Estudios Para el Desarrollo Sustentable de Zonas Costeras, Centro Universitario de la Costa Sur Universidad de Guadalajara Melaque Jalisco Mexico; ^4^ Department Evolutionary Ecology Leibniz Institute for Zoo and Wildlife Research Berlin Germany

**Keywords:** diet, seabird, sexual segregation, stable isotope analysis, tropics

## Abstract

Animals that co‐occur in a region (sympatry) may share the same environment (syntopy), and niche differentiation is expected among closely related species competing for resources. The masked booby (*Sula dactylatra*) and smaller congeneric red‐footed booby (*Sula sula*) share breeding grounds. In addition to the inter‐specific size difference, females of both species are also larger than the respective males (reversed sexual size dimorphism). Although both boobies consume similar prey, sometimes in mixed‐species flocks, each species and sex may specialize in terms of their diet or foraging habitats. We examined inter‐ and intra‐specific differences in isotopic values (δ^13^C and δ^15^N) in these pelagically feeding booby species during the incubation period at Clarion Island, Mexico, to quantify the degrees of inter‐ and intra‐specific niche partitioning throughout the annual cycle. During incubation, both species preyed mainly on flyingfish and squid, but masked boobies had heavier food loads than red‐footed boobies. There was no overlap in isotopic niches between masked and red‐footed boobies during breeding (determined from whole blood), but there was slight overlap during the non‐breeding period (determined from body feathers). Female masked boobies had a higher trophic position than conspecific males during breeding; however, no such pattern was detected in red‐footed boobies. These results provide evidence of inter‐ and intra‐specific niche partitioning in these tropical seabird species, particularly during the breeding period and in the more‐dimorphic species. Our results suggest that these closely related species use different strategies to cope with the same tropical marine environment.

## Graphical abstract

Inter‐ and intra‐specific niche partitioning in tropical seabird species during the breeding period and in the more‐dimorphic species.

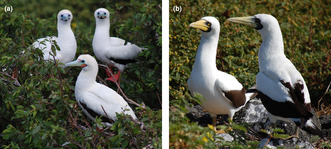

## 1 INTRODUCTION

An ecological niche defines the multi‐dimensional space of biotic and abiotic conditions that comprise the habitat or resource requirements of an organism (Chase & Leibold, 2003; Newsome et al., 2007). Species that occur in the same region (sympatry) and share habitats at the same time (syntopic) may compete for limited resources, especially closely related species with similar morphological traits (Gause, 1934; Hart et al., 2018; Shealer, 2002; Tanner et al., 2023). Theoretically, two species with identical ecological niches cannot coexist within the same habitat, and partitioning on at least some dimensions of the trophic niche, such as diet, space, or time, has been documented in many marine species, such as seabirds (Cherel et al., 2008; Navarro et al., 2015; Shealer, 2002).

Niche partitioning can occur spatially (Ashmole, 1971; Navarro et al., 2013), temporally (Kronfeld‐Schor & Dayan, 2003), or by diet (Robertson et al., 2014; Shealer, 2002), and occur both among and within species. Examples of intra‐specific niche partitioning are particularly common in species with sexual size dimorphism (Lerma, Dehnhard, et al., 2020; Mancini et al., 2013; Phillips et al., 2017). Inter‐ and intra‐specific differences are often linked to differences in body size, with larger individuals or species dominating in areas with higher prey availability (Catry et al., 2005; Phillips et al., 2017; Selander, 1966), using different foraging areas (Shoji et al., 2023; Weimerskirch et al., 2009; Zavalaga et al., 2007, 2010), or feeding on a greater range of prey sizes (Cohen et al., 1993; Mancini & Bugoni, 2014). In contrast, smaller individuals or species might be more agile, travel longer distances, or specialize in smaller prey, thanks to their lower energetic constraints (Ballance et al., 1997; Mancini et al., 2014; Shoji et al., 2023; Weimerskirch et al., 2006).

Tropical oceanic areas are considered to have relatively low productivity and a more patchy and less predictable distribution of resources compared with polar and temperate areas, but still host large colonies of seabirds (Ballance et al., 1997; Longhurst & Pauly, 1987; Weimerskirch, 2007). Tropical seabird species are often limited to foraging at or near the sea surface (Ashmole, 1971; Shealer, 2002), potentially leading to intense competitive exclusion (Ballance et al., 1997). Nevertheless, many tropical seabird species rely on similar prey items, use other birds as information cues to detect prey, and forage in mixed‐species flocks (Ashmole, 1971; Ballance et al., 1997; Spear et al., 2007; Thiebault et al., 2014; Veit & Harrison, 2017), leading to questions about the degree to which tropical seabird species are able to coexist and practice niche partitioning. Little evidence for trophic segregation has been found for some species of fish (Teffer et al., 2015), sharks (Lear et al., 2021), cetaceans (Peters et al., 2022), and seabirds (Forero et al., 2004; Petalas et al., 2024; Weimerskirch et al., 2009), and this was attributed to food being sufficiently abundant to allow species to coexist, at least during specific periods of the year.

Stable isotopes are a useful tool for evaluating inter‐ and intra‐specific niche differences, because the isotopic composition of the tissues reflects the isotopic composition of their assimilated prey. Blood samples can provide information on the diet assimilated during the previous 3–4 weeks (Vander Zanden et al., 2015), whereas body feathers give information about the diet during the period of formation (from weeks to months; Grecian et al., 2015; Petalas et al., 2024), which usually occurs during the non‐breeding period. Nitrogen isotopes (δ^15^N) increase predictably from prey to predator and are a useful proxy for the trophic position of the organism (DeNiro & Epstein, 1981; Hobson & Clark, 1992), while carbon isotopes (δ^13^C) increase predictably from inshore to offshore food webs (Cherel & Hobson, 2007). The isotopic niche and its dimensions have thus been used to study the trophic ecology and niches of several marine predators including fish (Kojadinovic et al., 2008; Teffer et al., 2015), sharks (Lear et al., 2021), cetaceans (Peters et al., 2022), and many species of seabirds (Kojadinovic et al., 2008; Navarro et al., 2015; Robertson et al., 2014; Shoji et al., 2023).

**FIGURE 1:**
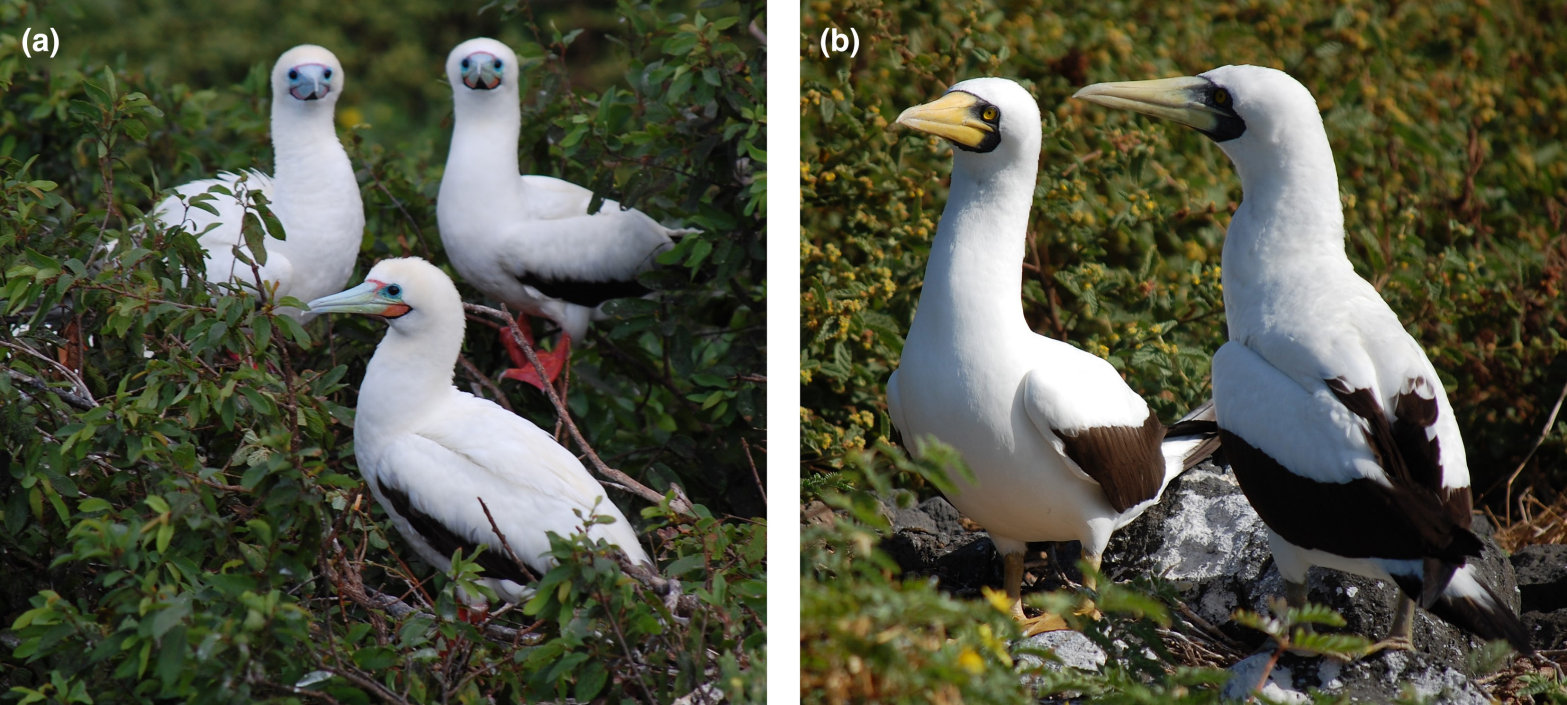
Red‐footed boobies (*Sula sula*) (a) and masked boobies (*Sula dactylatra*) (b) occur sympatrically at Clarion Island, Revillagigedo Archipelago, Mexico.

Boobies (*Sula* spp.) are ideal species for understanding the prevalence of inter‐ and/or intra‐specific trophic segregation in tropical areas. Masked boobies (*Sula dactylatra*, Lesson 1831) and red‐footed boobies (*Sula sula*, Linnaeus 1766) (Figure 1) have a pantropical distribution and often share breeding grounds (Kappes et al., 2011; Nelson, 1978; Young, Shaffer, et al., 2010). Both booby species prey mostly on flyingfish and squid throughout their ranges (Donahue et al., 2020; Kappes et al., 2011; Lerma, Dehnhard, et al., 2020; Schreiber & Hensley, 1976; Young, McCauley, et al., 2010) and may form mixed‐species flocks (Ballance et al., 1997; Spear et al., 2007). Both species also show reversed sexual size dimorphism, with females being larger than males; however, masked boobies are notably more dimorphic than red‐footed boobies (Nelson, 1978; Van Oordt et al., 2018). Examples of, and exceptions to, inter‐ and intra‐specific niche segregation are common in boobies and have been found in some, but not other colonies (see Tables 1 and 2). The contrasting results within species and colonies may be associated with the distribution of their prey, with the study period, or with the size of the colonies. For example, foraging segregation might be more common in heterogeneous (Castillo‐Guerrero et al., 2016) than in homogeneous environments (Lerma, Serratosa, et al., 2020), during breeding (when birds are limited to foraging close to their colonies) compared with non‐breeding periods (Phillips et al., 2017; Roy et al., 2021), and in larger colonies compared to smaller ones (Austin et al., 2021; Petalas et al., 2024; Soanes et al., 2016; Wakefield et al., 2017).

**TABLE 1:** Inter‐specific foraging differences in sympatrically breeding booby species.

	Red‐footed	Masked	Abbots	Nazca	Brown	Blue‐footed
Masked	≠^T1,T2,T3^					
Abbott's	NC	U^C1^				
Nazca	U^C2^	U^C2^	NC			
Brown	≠^T1,T4,T5^	≠^T1,C1,C3^	U^C1^	NC		
Blue‐footed	U^C4^	NC	NC	≠^T7^	≠^T6^	
Peruvian	NC	NC	NC	NC	NC	U^C5^

*Note*: Only studies that evaluated either foraging parameters and/or stable isotopes were included in this comparison.

Abbreviations: ≠, inter‐specific differences were found; NC, not co‐occurring; U, undetermined.

^T1^Tested on Palmyra atoll: red‐footed boobies traveled farther from the colonies than masked boobies, δ^13^C was more depleted in masked boobies than in red‐footed boobies (Young, McCauley, et al., 2010; Young, Shaffer, et al., 2010).

^T2^Tested on Tromelin Island: masked boobies traveled farther from the colonies and consumed larger prey than red‐footed boobies (Kappes et al., 2011).

^T3^Tested on Clipperton Island: δ^15^N values were higher and δ^13^C was more depleted in masked boobies than in red‐footed boobies (Bustamante et al., 2023).

^T4^Tested on Cabo Verde: red‐footed boobies traveled farther from the colonies than brown boobies and there were isotopic niche difference between species (Almeida et al., 2021).

^T5^Tested on Cayman Islands: red‐footed boobies traveled farther offshore than brown boobies (Austin et al., 2021).

^T6^Tested on Baja California: no difference in foraging range or isotopic niches between blue‐footed boobies and brown boobies, but blue‐footed boobies dived deeper than brown boobies (Weimerskirch et al., 2009).

^T7^Tested on Galapagos: Nazca boobies (formerly masked) made longer trips than blue‐footed boobies (Anderson & Ricklefts, 1992).

Co‐occurring but inter‐specific foraging differences have not been tested: ^C1^Christmas Island, ^C2^Galapagos Islands, ^C3^Abrolhos, Atol das Rocas, and Fernando de Noronha, ^C4^Isla Isabel, ^C5^Lobos de Tierra.

**TABLE 2:** Intra‐specific isotopic niche segregation in masked and red‐footed boobies.

	Blood	Feathers	References
Red‐footed booby			
Clarion Island^a^	F = M	F = M	This study
Palmyra Atoll^b^	F = M	F = M	Young, McCauley, et al. (2010)
Raine Island^c^	F = M	F = M	Pontón‐Cevallos et al. (2017)
Europa Island^d^	F ≠ M	F = M	Cherel et al. (2008)
Cayman Islands^e^	F ≠ M		Austin et al. (2021)
Clipperton Island^a^	F = M		Bustamante et al. (2023)
Cabo Verde^f^	F = M		Almeida et al. (2021)
Xisha Islands^g^		U	Wu et al. (2018)
Masked booby			
Clarion Island^a^	F ≠ M	F = M	This study
Palmyra Atoll^b^	F = M	F = M	Young, McCauley, et al. (2010)
Rapa Nui^c^	F ≠ M		Lerma, Serratosa, et al. (2020)
Clipperton Island^a^	F = M		Bustamante et al. (2023)
Abrolhos^h^	F = M		Mancini et al. (2013)
Atol das Rocas^h^	F = M		Mancini et al. (2013)
Fernando de Noronha^h^	F = M		Mancini et al. (2013)

*Note*: Only studies were stable isotopes where evaluated are included.

Abbreviations: F, female; M, male.

^a^ Eastern Pacific Ocean.

^b^ Central Pacific.

^c^ South Pacific Ocean.

^d^ Indian Ocean.

^e^ Caribbean Sea.

^f^ Central Atlantic Ocean.

^g^ South China Sea.

^h^ South Atlantic Ocean.

Both masked and red‐footed boobies breed at Clarion Island (Almanza‐Rodríguez, 2019; Wanless et al., 2009), which is surrounded by an oligotrophic environment (Lerma, Castillo‐Guerrero, et al., 2020). The waters adjacent to Clarion Island show no extreme environmental variations throughout the year (Lerma, Castillo‐Guerrero, et al., 2020), and some masked and red‐footed boobies can be found at the island, regardless of the time of year (Almanza‐Rodríguez, 2019; Brattstrom & Howell, 1956; Everett, 1988; Wanless et al., 2009). The year‐round occurrence of tropical seabird species close to their colonies has been attributed to low but stable prey abundances, which might offer continuous foraging and breeding opportunities, but only for a limited number of individuals (Almeida et al., 2021; Lerma, Serratosa, et al., 2020; Roy et al., 2021). Recent studies accordingly showed that both masked (Roy et al., 2021) and red‐footed boobies (Votier et al., 2023) can be resident species.

We aimed to investigate the degrees of inter‐ and intra‐specific trophic segregation in masked and red‐footed boobies during the breeding and non‐breeding period. We collected and analyzed diet samples, and measured δ^13^C and δ^15^N values in blood and feather samples. Whole blood indicates the food assimilation during the incubation and/or pre‐laying period, whereas body feathers, during the non‐breeding period. We had four main predictions. First, masked boobies, as the larger species, were expected to consume more‐diverse and larger prey items (Kappes et al., 2011; Young, Shaffer, et al., 2010), resulting in higher δ^15^N values and wider trophic niche (Queirós et al., 2021; Wu et al., 2017) compared with red‐footed boobies. However, we did not have clear predictions for δ^13^C values, because both species have been reported to forage in mixed flocks (Ballance et al., 1997; Spear et al., 2007), or masked boobies have been found to forage closer to their colonies than red‐footed boobies in some studies (Young, Shaffer, et al., 2010), while the opposite occurred in other studies (Kappes et al., 2011). Second, sexual size dimorphism in both booby species should enable the larger females to feed on more diverse and larger prey, and we therefore predicted that females would exhibit higher δ^15^N values than in males in both species. Furthermore, males are expected to travel farther from their colonies, and we therefore predicted lower δ^13^C values in females than in males in both species. Third, we expected that higher competition and constraints in foraging areas during the breeding period, due to central‐place foraging, would lead to greater differences both within and between species, and therefore predicted that whole blood samples would show pronounced differences in niche width (variety of resources consumed) and niche position (types of resources consumed), and lower niche overlap (similarity in resource use). In contrast, lower competition and less constraint in foraging areas would lead to greater niche overlap between booby species and sexes during the non‐breeding period. Fourth, given that masked and red‐footed boobies might be resident species, we predicted that each species would have relatively similar δ^15^N and δ^13^C values in their breeding and non‐breeding periods.

## 2 METHODS

### 2.1 Sample collection

**FIGURE 2:**
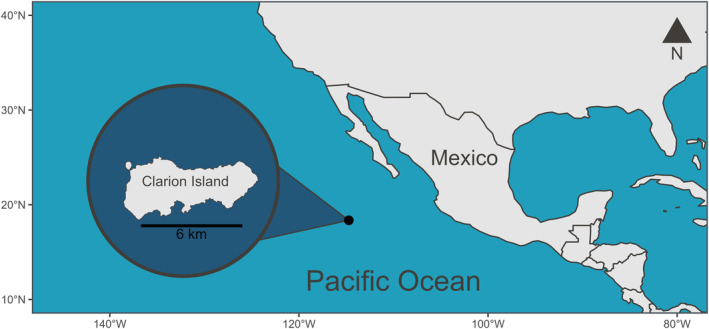
Location of Clarion Island, part of the Revillagigedo Archipelago, Mexico, in the Pacific Ocean. On Clarion Island, the masked booby (*Sula dactylatra*) colony includes <100 breeding pairs and the red‐footed booby (*Sula sula*) colony includes >3000 breeding pairs. In order to study variations in inter‐specific and sex‐related niche partitioning, whole blood and body feathers from a total of 36 masked and 22 red‐footed boobies were collected during March 2017 and 2018.

This study was conducted at Clarion Island, Revillagigedo Archipelago, Mexico (18°21′7.53″ N, 114°43′18.61″ W; Figure 2), in March 2017 and March 2018. The island lies 985 km from the Mexican mainland and is 710 km southwest of the Baja California Peninsula (Wanless et al., 2009), situating the island far from the coastal upwelling. The environmental conditions within the foraging ranges of boobies (<180 km) did not differ significantly between March 2017 and March 2018: chlorophyll concentrations average 0.09 ± 0.02 mg/m^3^ and sea surface temperature averages 26.3 ± 1.3°C in both years (Lerma, Castillo‐Guerrero, et al., 2020). The red‐footed booby colony includes >3000 breeding pairs and the masked booby colony includes <100 breeding pairs (Almanza‐Rodríguez, 2019; Wanless et al., 2009). Other seabird species breeding on Clarion Island include Nazca boobies (*Sula granti*, two pairs), Brown boobies (*S. leucogaster*, eight pairs), Laysan Albatross (*Phoebastria immutabilis*, 46 pairs), and Red‐billed tropicbirds (*Phaethon aethereus*, 48 pairs) (Almanza‐Rodríguez, 2019; Wanless et al., 2009).

Diet samples were collected opportunistically from masked and red‐footed boobies that regurgitated spontaneously as a result of our presence in the colony or during manipulation. A total of 34 samples were collected in March 2017 and 38 in March 2018. From this, 59 diet samples were from masked boobies (prey items = 221) and 13 from red‐footed boobies (prey items = 36). Due to the opportunistic method of sample collection, the sex of the bird was not recorded. The whole regurgitate was placed in an individual plastic bag and weighed to the nearest 1 g. Each prey item was then removed from the bag, measured to the nearest 1 mm using a ruler, and photographed for subsequent reference. Due to digestion, prey samples were only identified to family level, based on Pacific fish guides (Fischer et al., 1995). The samples were collected at night, and given that boobies are diurnal feeders (Nelson, 1978), night sampling might have unintentionally led to a higher proportion of partly digested items, thus preventing the detection of small or soft prey items (Barrett et al., 2007). This can be particularly important for detecting differences in dietary analysis in red‐footed boobies, which are known to prey on a higher proportion of squid (Donahue et al., 2020).

Individual masked and red‐footed boobies were captured at their nest by hand or using a hand net from a distance of 1–2 m. Masked boobies were captured at their nest on the ground, and red‐footed boobies were captured at their nest in the bushes. Individuals were captured between 19:00 and 03:00 h to prevent sunstroke to the birds and to avoid potential predation of eggs and chicks by common ravens (*Corvus corax*) or Clarion Island whip snakes (*Masticophis anthonyi*). Captured individuals were incubating, and most nests were confirmed to contain eggs. A total of 42 individuals were captured in March 2017 (32 masked boobies and 10 red‐footed boobies) and 39 in 2018 (26 masked boobies and 13 red‐footed boobies). Sex was determined based on size and vocalizations in masked boobies and confirmed using molecular markers in red‐footed boobies. The total handling time never exceeded 10 min, to minimize distress to the birds. All captured individuals were measured using Vernier calipers (±0.01 mm) and weighed using a digital balance (±1 g). Masked boobies were 38% heavier and 17%–38% larger than red‐footed boobies (Table 3). The degree of dimorphism was greater in masked boobies and almost absent in red‐footed boobies: in masked boobies, females were 10% heavier and 1.9%–4.5% larger than males, whereas in red‐footed boobies females were 5% heavier and 0.3%–0.4% larger than males (Table 3).

**TABLE 3:** Body measurements of breeding masked boobies (*Sula dactylatra*, 28 females, 30 males) and red‐footed boobies (*Sula sula*, nine females, 14 males) at Clarion Island, Mexico.

	Masked booby				Red‐footed booby			
	Female	Male	Both	%	Female	Male	Both	%
Body mass (g)	2150 ± 250	1900 ± 180	2040 ± 240	10.0	1330 ± 77	1260 ± 250	1270 ± 180	5.0
Culmen (mm)	110 ± 5	107 ± 4	108 ± 4	1.9	90 ± 3	90 ± 6	90 ± 4	0.4
Tarsus (mm)	64 ± 3	61 ± 2	62 ± 3	4.5	44 ± 2	44 ± 6	43 ± 4	0.3

From all captured individuals, a total of 26 individuals were sampled in March 2017 (16 masked boobies and 10 red‐footed boobies) and 32 individuals in 2018 (20 masked boobies and 12 red‐footed boobies). A few drops of blood (~0.15 mL) were collected from the brachial vein of individual birds using a 25 G needle and non‐coated capillary tubes. The blood samples were placed on glass microscope slides and air‐dried. Whole blood reflects the diet assimilated during the previous 3–4 weeks (Vander Zanden et al., 2015), and whole blood samples therefore provide information on the bird's diet during the incubation and/or pre‐laying period. Body feathers were collected from the ventral part of adult birds and stored in individual paper bags. Body feathers were considered optimal given that they are easy to collect and do not impair the flight ability of the sampled birds (Bighetti et al., 2022; Jaeger et al., 2009). In contrast to whole blood, body feathers integrate information about the diet during the period when the feather was formed (from weeks to months; Grecian et al., 2015; Petalas et al., 2024), which is usually during the non‐breeding period for these boobies (Grace et al., 2020; Schreiber et al., 2020). As mentioned before, no signs of molting, such as missing primary feathers, worn feathers of feather growth were observed while manipulating the individuals, supporting that molting occurs outside the breeding season.

### 2.2 Laboratory analyses

Dried whole blood samples (0.2–0.6 mg) were scraped from the slides and placed in tin cups in the laboratory. The feathers were immersed in a 2:1 chloroform and methanol solvent to remove surface oils and associated contaminants (Hobson et al., 2014). The samples did not undergo lipid extraction, and the low C:N (all <4) mass ratios indicated that mathematical correction for high lipid content was not required (Post et al., 2007). The isotope values of all samples were analyzed at the Leibniz Institute for Zoo and Wildlife Research, Berlin, Germany, using a Flash elemental analyzer (Thermo Fisher Scientific, Bremen, Germany) connected in sequence via a ConFlo (Thermo Fisher Scientific) to a stable isotope ratio mass spectrometer (Delta V; Thermo Fisher Scientific). The instrument was flushed with chemically pure helium gas for measurements. Stable isotope ratios were expressed in delta notation indicating the deviation from international standards (in air nitrogen for nitrogen and V‐PDB for carbon), according to the equation: δX = [(*R*_sample_/*R*_standard_) − 1], where X is ^13^C or ^15^N and R is the ratio ^13^C/^12^C or ^15^N/^14^N, respectively. Secondary isotopic reference materials were tyrosine (δ^13^C: −23.96 ± 0.02‰; δ^15^N: 4.36 ± 0.04‰) and leucine (δ^13^C: −30.15 ± 0.05‰; δ^15^N: 10.82 ± 0.08‰). The analytical precision of both δ^13^C and δ^15^N was of <0.2‰.

### 2.3 Statistical analyses

Using a total of 59 diet samples collected from masked boobies (prey items = 221) and 13 from red‐footed boobies (prey items = 36), the relative frequency of occurrence, diet composition, food‐load mass, and length of prey items were compared between species. The relative frequency of occurrence was defined as the percentage of birds with a particular species in their diet sample. The diet composition according to prey families was compared between the booby species by a χ^2^ test, and the food‐load mass was compared using *t*‐tests. Food‐load mass included partly digested and undigested items and was only an approximation. The length of the prey items was also compared using *t*‐tests, but only included prey items that were complete from head to tail. Among a total of 257 samples, only 16% were complete, 34 samples from masked boobies and seven from red‐footed boobies.

We investigated inter‐ and intra‐specific differences in masked and red‐footed boobies between the breeding and non‐breeding periods based on stable isotope data. First, niche width was analyzed as a proxy for the variety of resources consumed and was evaluated using a test for differences in dispersion following Turner et al. (2010), which measures the average trophic variability within groups. Using analyses of nested linear models and a residual permutation procedure, the mean distance to the centroid was calculated per group, and the absolute value of the difference was evaluated between groups (Hammerschlag‐Peyer et al., 2011). Second, the niche position was used as a proxy of the types of resources consumed and was measured by computing the Euclidean distance between the centroids of the groups (Turner et al., 2010). Third, niche overlap was used as a proxy of similarity of resource use and was calculated using the function: [area of overlapping region]/([area of ellipse 1] + [area of ellipse 2] − [area of overlapping region]) using standard ellipse areas adjusted for small sample sizes calculated using the package SIBER (Jackson et al., 2011). Differences between the breeding and non‐breeding periods were determined using body feathers for the non‐breeding period and standardized whole blood values for the breeding period. A mathematical correction was applied to standardize whole blood δ^13^C and δ^15^N to make it comparable with isotope values in feathers (Cherel et al., 2014). The equation to standardize whole blood is as follows: δ^13^C_feather_ = 0.972 (±0.020) δ^13^C_blood_ + 0.962 (±0.414) and δ^15^N_feather_ = 1.014 (±0.056) δ^15^N_blood_ + 0.447 (±0.414). This equation allows to account for blood being impoverished in ^13^C and ^15^N compared with feathers. Differences in bulk δ^13^C and δ^15^N values were additionally compared using Tukey's HSD tests. Due to numerous combinations of species, sex, and periods, comparisons between years in niche width, niche position, and niche overlap would have resulted in small sample sizes and were therefore omitted. Moreover, as already indicated, the environmental conditions between the years of the study were similar (Lerma, Castillo‐Guerrero, et al., 2020). All statistical analyses were performed in R 4.0.3 (R Core Team, 2023), and an alpha of 0.05 was used as the threshold for significance.

## 3 RESULTS

### 3.1 Diet

**FIGURE 3:**
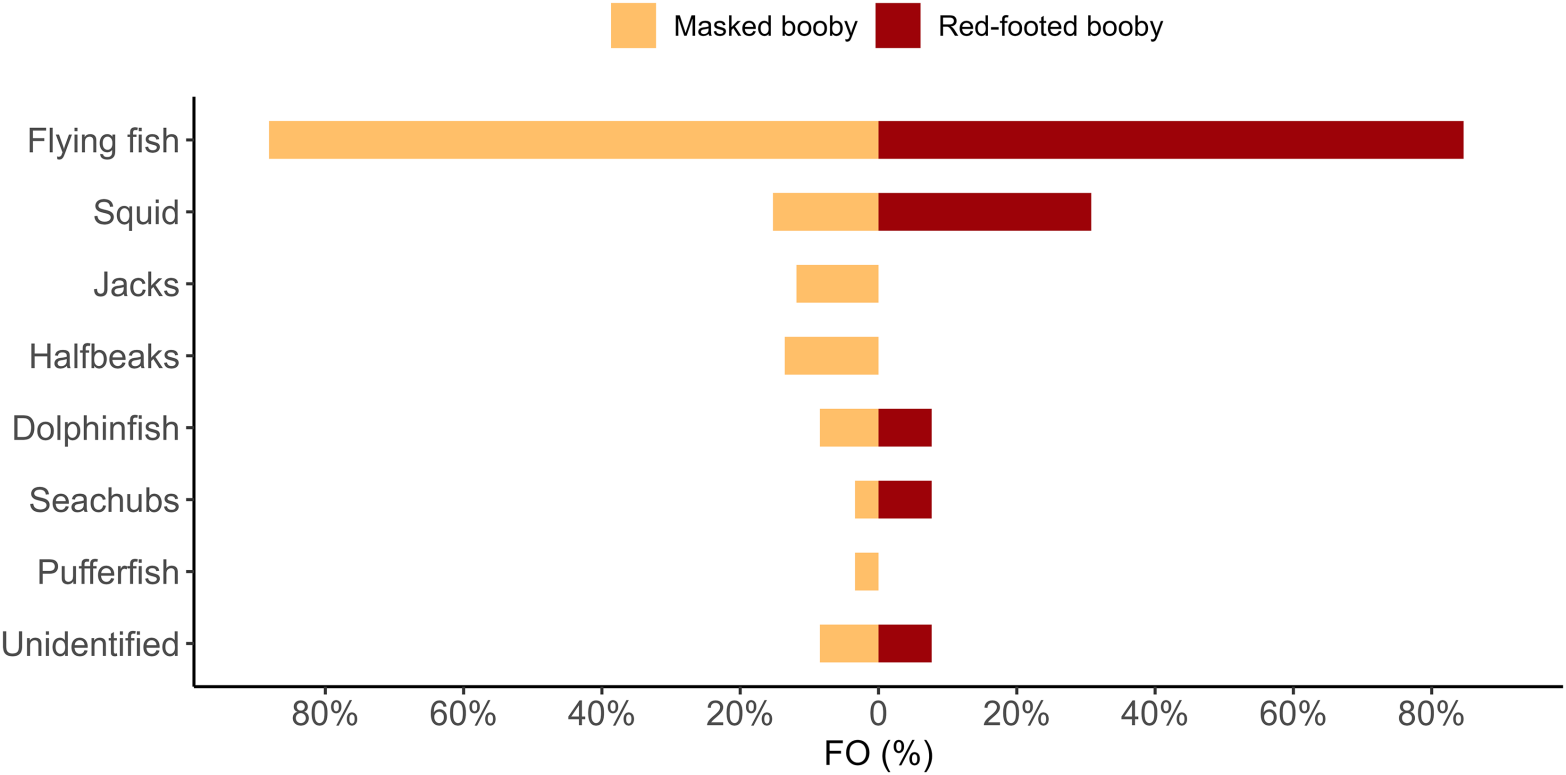
Relative frequency of occurrence (FO%) of prey items in diet samples from masked boobies (*Sula dactylatra*, *n* = 59) and red‐footed boobies (*Sula sula*, *n* = 13) on Clarion Island, Revillagigedo Archipelago, Mexico, in the Pacific Ocean. Families identified included flyingfish (Exocoetidae), squid (Omnastrephidae), jacks (Carangidae), halfbeaks (Hemiramphidae), dolphinfish (Coryphaenidae), seachubs (Kyphosidae), and pufferfish (Tetraodontidae).

Flyingfish (Exocoetidae) was the main prey item for both masked and red‐footed boobies with more than 80% of frequency of occurrence in both species (Figure 3). Masked boobies had a more diverse diet than red‐footed boobies by including jacks (Carangidae), halfbeaks (Hemiramphidae), and pufferfish (Tetraodontidae), whereas red‐footed boobies included a higher proportion of squid in their diet than masked boobies (Figure 3). Masked booby diet samples contained an average of 3.7 ± 2.4 items from one to five families, and red‐footed booby diet samples contained 2.8 ± 2.4 items from one to two families. The proportion of prey items in the diet according to family was homogeneous between species (${\chi^{2}}_{6}$ = 10, *p* = .12). The food‐load mass for masked boobies was 149.5 ± 88.7 g, while that for red‐footed boobies was almost half that weight (87.5 ± 65.3 g) (*t*‐test = 2.56, *p* = .02). The average prey length for masked boobies was 15.9 ± 8.3 mm (max 33.7 cm) and that for red‐footed boobies was 13.1 ± 3.4 mm (17.0 cm), with no significant difference between the species (*t*‐test = 1.47, *p* = .15).

### 3.2 Stable isotopes

#### 3.2.1 Breeding period

**FIGURE 4:**
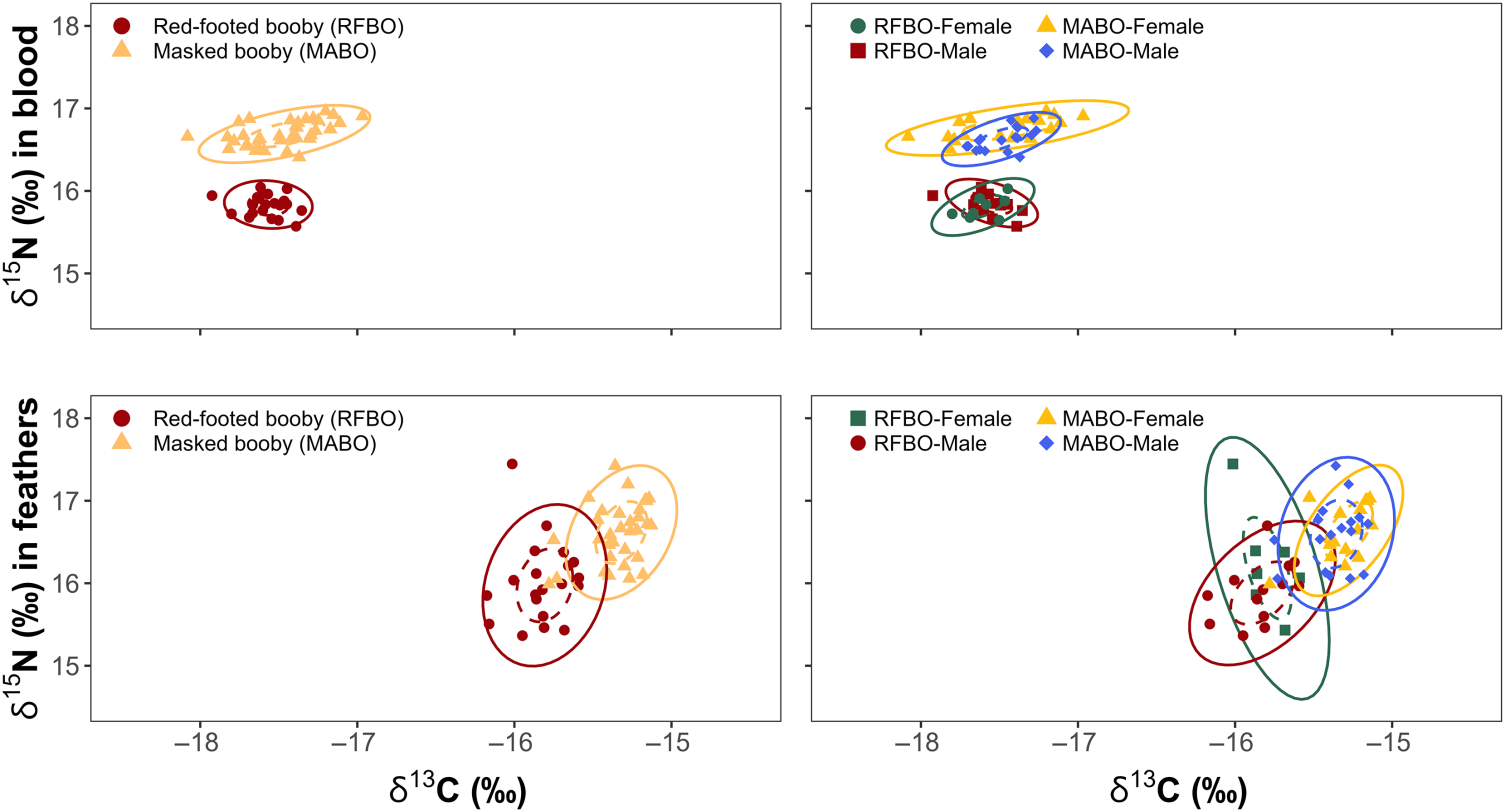
Isotopic niche ellipses from masked boobies (*Sula dactylatra*) and red‐footed boobies (*Sula sula*) on Clarion Island, Revillagigedo Archipelago, Mexico, in the Pacific Ocean during March 2017 and 2018. Upper panels: whole blood (breeding) from masked (females = 18, males = 18) and red‐footed boobies (females = 8, males = 14). Lower panels: body feathers (non‐breeding) from masked (females = 15, males = 18) and red‐footed boobies (females = 7, males = 13). Standard ellipses of 50% are depicted as dotted and 95% ellipses as complete ellipses. Points represent individual measurements.

**FIGURE 5:**
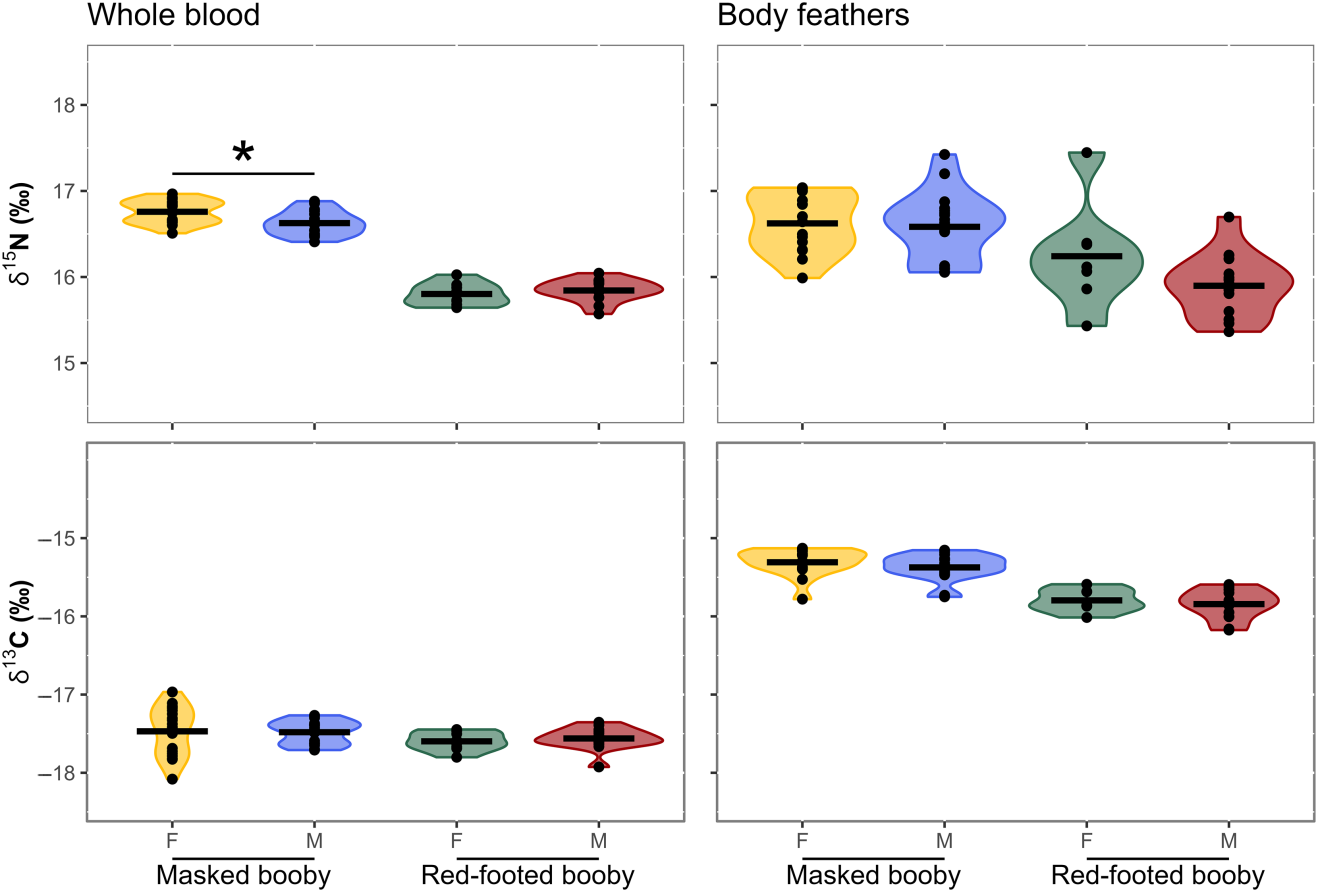
Violin plots of δ^13^C and δ^15^N values in samples from masked (*Sula dactylatra*) and red‐footed boobies (*Sula sula*) on Clarion Island, Revillagigedo Archipelago, Mexico, in the Pacific Ocean during March 2017 and 2018. Left panels: whole blood (breeding) from masked boobies (females = 18, males = 18) and red‐footed boobies (females = 8, males = 14). Right panels: body feathers (non‐breeding period) from masked boobies (females = 15, males = 18) and red‐footed boobies (females = 7, males = 13). Dots represent individual measurements, lines represent mean values, and asterisk indicates significant differences between sexes.

Masked and red‐footed boobies showed inter‐specific niche partitioning during the breeding period. The niche width (based on δ^13^C and δ^15^N isotope values) differed between species (mean distance to centroid = 0.09, *p* < .01), and the Euclidian distance between centroid locations (taking both δ^15^N and δ^13^C values) also differed significantly between the species (*p* < .01), with no niche overlap (Figure 4). As predicted, δ^15^N values were significantly higher in masked than in red‐footed boobies (Tukey's HSD < 0.01), but there was no significant difference in δ^13^C values (Tukey's HSD = 0.08) (Figure 5).

Masked boobies showed intra‐specific differences during the breeding period. Females and males did not differ in their niche width (mean distance to centroid = 0.11, *p* = .16) and their isotopic niche areas overlapped (<33% overlap) (Figure 4), but the Euclidian distance between centroid locations (niche position) differed significantly between the sexes (*p* < .01). The δ^15^N values were higher in females than in males (Tukey's HSD < 0.01), whereas the δ^13^C values (Tukey's HSD = 0.88) were not significantly different.

There were no intra‐specific differences in red‐footed boobies during breeding. Females and males did not differ in terms of niche width (mean distance to centroid <0.01, *p* = .67), or Euclidian distance between centroid locations (*p* = .60), and there was high overlap in their isotopic niche areas (<46% overlap) (Figure 4). Moreover, the δ^13^C and δ^15^N values for female and male red‐footed boobies were not significantly different (Tukey's HSD > 0.05).

#### 3.2.2 Non‐breeding period

Masked and red‐footed boobies showed niche partitioning during the non‐breeding period, with significant differences in niche width between species (mean distance to centroid = 0.06, *p* < .01) and the Euclidian distance between centroid locations (*p* < .01). Masked boobies had significantly higher δ^15^N and δ^13^C values than red‐footed boobies (Tukey's HSD < 0.01), suggesting that masked boobies generally foraged on different prey and/or in different areas than red‐footed boobies during the non‐breeding period. However, the standard ellipse areas showed some overlap (<0.16% overlap) (Figure 4), suggesting that both species shared at least some subset of prey diversity during the non‐breeding period.

During the non‐breeding period, female and male masked boobies showed no significant difference in niche width (mean distance to centroid = 0.02, *p* = .62), or Euclidian distance between centroid locations (*p* = .49), and there was high niche overlap (<67% overlap) (Figure 4). There was also no difference in δ^15^N and δ^13^C values between females and males masked boobies (Tukey's HSD > 0.05) (Figure 5).

Similarly, during the non‐breeding period, female and male red‐footed boobies showed no differences in niche width (mean distance to centroid = 0.11, *p* = .12), or Euclidian distance between centroid locations (*p* = .29), and there was niche overlap (<38% overlap) (Figure 4). Although δ^15^N appeared to be higher in non‐breeding female than male red‐footed boobies, the differences in δ^15^N (Tukey's HSD = 0.13) and δ^13^C (Tukey's HSD = 0.56) were not significant.

#### 3.2.3 Breeding versus non‐breeding period

**FIGURE 6:**
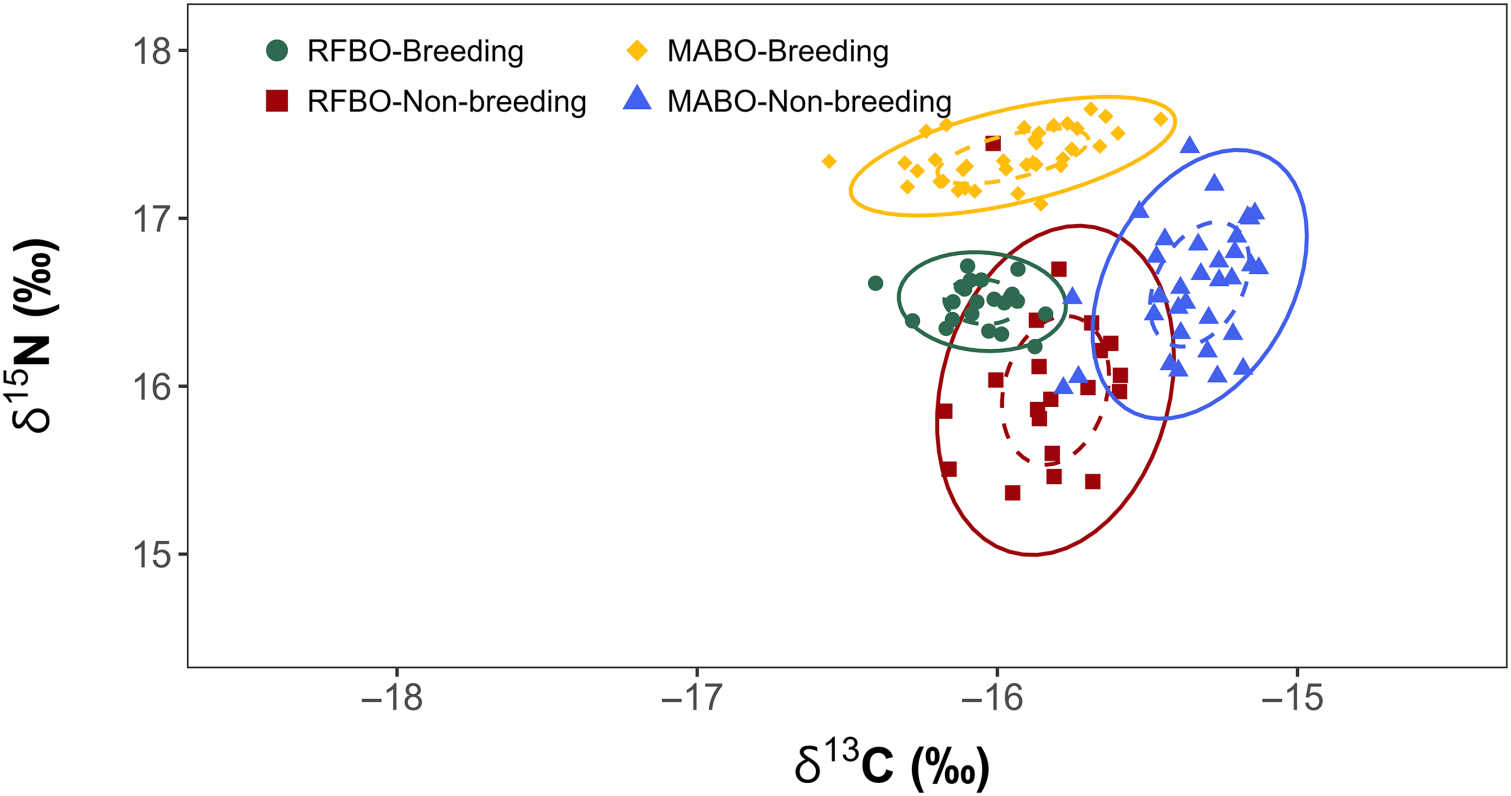
Isotopic niche ellipses from masked boobies (*Sula dactylatra*) and red‐footed boobies (*Sula sula*) on Clarion Island, Revillagigedo Archipelago, Mexico, in the Pacific Ocean during March 2017 and 2018. Standardized whole blood (breeding) from masked boobies (*n* = 36) and red‐footed boobies (*n* = 22) and body feathers (non‐breeding) from masked boobies (*n* = 33) and red‐footed boobies (*n* = 20) were included in the analyses. Standard ellipses of 50% are depicted as dotted and 95% ellipses as complete ellipses. Points represent individual measurements.

In masked boobies, niche width differed significantly between the breeding and non‐breeding periods (mean distance to centroid = 0.09, *p* < .01). The Euclidian distance between centroid locations showed differences in niche positions between these periods (*p* < .01), and there was no niche overlap (Figure 6). Body feathers showed significantly higher δ^13^C and lower δ^15^N values than standardized whole blood (Tukey's HSD < 0.01 for both).

For red‐footed boobies, niche width (mean distance to centroid = 0.24, *p* = .01) and Euclidian distance between centroid locations also differed significantly between the breeding and non‐breeding periods (*p* < .01). Red‐footed boobies showed significantly lower δ^15^N values in body feathers than in standardized whole blood (Tukey's HSD < 0.01), suggesting that they consumed different prey during the breeding and the non‐breeding periods. However, there was niche overlap (<14% overlap) (Figure 6), indicating that at least a subset of their general prey was used during both periods. Moreover, δ^13^C values were not significantly different (Tukey's HSD = 0.99), suggesting that red‐footed boobies might use similar foraging habitats year‐round.

## 4 DISCUSSION

In the present study, we had four main predictions. First, we found that breeding masked and red‐footed boobies consumed items from similar prey families at Clarion Island, but in line with our predictions, the food‐load mass was heavier in masked than in red‐footed boobies. Despite preying on similar families though, there were differences in niche position and niche overlap between the two booby species during the breeding period. After matching differences in food‐load mass, masked boobies had higher δ^15^N values than red‐footed boobies, as expected, while δ^13^C values were similar. Second, we found evidence of intra‐specific resource partitioning, as expected, but this only occurred in masked boobies during the breeding period, and not during the non‐breeding period, and did not occur in red‐footed boobies in either period. This agreed with our expectation that sexual size dimorphism might play a role in promoting resource partitioning, but it was only relevant during the breeding period and in the more‐dimorphic species. Third, differences between the species in niche position also occurred during the non‐breeding period, but there was some overlap, supporting our expectation that differences would be greater during the breeding than during the non‐breeding period. Fourth, we found larger differences between the breeding and the non‐breeding period in masked than in red‐footed boobies, suggesting that red‐footed boobies foraged in similar areas year‐round, as predicted, whereas masked boobies moved their foraging areas between the breeding and non‐breeding period.

### 4.1 Diet

In agreement with the literature, flyingfish and squid were the main prey items in the diets of both masked and red‐footed boobies (Donahue et al., 2020; Kappes et al., 2011; Lerma, Dehnhard, et al., 2020; Schreiber & Hensley, 1976; Young, McCauley, et al., 2010). Aguilar Nuño (2019) found that oceanic two‐wing flyingfish (*Exocoetus obtusirostris*) and whitetip flyingfish (*Cheilopogon xenopterus*) dominated the diet of masked boobies at Clarion Island, whereas spotfin flyingfish (*Cheilopogon furcatus*) and oceanic two‐wing flyingfish (*Exocoetus obtusirostris*) were dominant in the diet of red‐footed boobies. In the current study, we were unfortunately unable to identify prey items to species level due to our sampling method. We also acknowledge that the current sample size was small (59 diet samples from masked boobies and 13 diet samples from red‐footed boobies), and thus, the importance of particular prey species and differences in prey length between booby species might have been underestimated. Nonetheless, we found that the overall food‐load mass was larger in masked than in red‐footed boobies, matching findings from Tromelin Island (Kappes et al., 2011) and in accordance with the larger size of masked boobies (38% heavier and larger than red‐footed boobies, see Table 3). Although there was no significant difference in the length of the prey items consumed by each booby species, we found that the maximum prey length for masked boobies was almost double that for red‐footed boobies. Ideally however, future studies should increase the sample size and/or include metagenomic analyses (Carreiro et al., 2022; Donahue et al., 2020) to account for partially digested items.

### 4.2 Inter‐specific differences

As expected, masked boobies had higher δ^15^N values than red‐footed boobies, in agreement with the literature (see Table 2 for references). The difference in δ^15^N values between booby species is likely an effect of niche specialization and of physiological differences associated with body size, which allows each species to reach different habitats and consume different resources. Here, as expected, the larger size of masked boobies compared with red‐footed boobies enabled them to feed on a greater range of prey species and sizes (Cohen et al., 1993; Mancini & Bugoni, 2014), which in turn had higher δ^15^N values (Queirós et al., 2021; Wu et al., 2017). In addition, the larger size of masked boobies might allow them to dive deeper in the water column (Zavalaga et al., 2007, 2010). Masked boobies have been shown to dive up to 5.5 m (Lerma, Castillo‐Guerrero, et al., 2020), whereas red‐footed boobies only reach 2.4 m (Weimerskirch et al., 2005), providing additional support for the inclusion of deeper‐water fish, such as pufferfish and jacks, in the masked boobies' diet. Although in other booby species the inclusion of deeper‐water fish has been associated with fishing discards (Mancini et al., 2023), it would be difficult to explain why one, but not the other booby species would be making use of fisheries discards. Moreover, the use of fishing discards by boobies is unlikely to occur at Revillagigedo, as most foraging trips are concentrated within the protected area (Lerma, Castillo‐Guerrero, et al., 2020) where fishing boats were not observed, and fishing is not allowed (DOF, 2017).

The similar δ^13^C values in masked and red‐footed boobies suggest that both species forage in areas with similar carbon sources during breeding. However, both species may have used distinct foraging areas, which were not reflected in the δ^13^C levels (Mancini et al., 2013), and thus our results should be interpreted with caution. By using only stable isotopes, it is challenging to discriminate between areas at the boobies' foraging scales since the isoscape may exhibit homogeneous δ^13^C values at a regional scale (Magozzi et al., 2017). Moreover, whole blood reflects the diet assimilated in the previous 3–4 weeks (Vander Zanden et al., 2015), and our samples might thus have included information from the early incubation and/or pre‐laying periods, when differences in foraging distances and area use may have been absent. Nevertheless, our results of masked and red‐footed boobies showing similar δ^13^C values are in accordance with the fact that both species forage in mixed‐species flocks (Ballance et al., 1997; Spear et al., 2007), but contrasts with the findings that red‐footed boobies traveled farther from their colonies than masked boobies (Young, Shaffer, et al., 2010), or remained closer to their colonies than masked boobies (Kappes et al., 2011) at other breeding sites.

In Clarion, we found no difference in δ^13^C values, whereas in Palmyra masked boobies had more depleted δ^13^C values and forage closer to their colony than red‐footed boobies, in contrast to Tromelin where red‐footed boobies remained closer to the colony than masked boobies (see Table 1). Previous studies have attributed inter‐specific differences in δ^13^C values and foraging ranges to competitive exclusion. However, the small colony size of masked booby to the ratio of red‐footed boobies pairs makes it unlikely that masked boobies could exclude red‐footed boobies from extensive areas close to their colony. At Clarion Island, the masked booby colony is much smaller (<100 breeding pairs) than that of red‐footed booby (>3000 breeding pairs), in Palmyra Atoll, only 50 masked booby pairs breed sympatrically with 2500 red‐footed booby pairs (Young, Shaffer, et al., 2010), and in Tromelin, 130 masked booby pairs breed sympatrically with 200 red‐footed booby pairs (Kappes et al., 2011). Alternatively, predator presence can have an impact on inter‐specific segregation. Booby species with chicks vulnerable to predation might perform shorter trips to reduce their absences at the nest (Anderson, 1991; Anderson & Ricklefts, 1992). However, this might not be the case for Clarion Island, as eggs and chicks of both, masked and red‐footed boobies, are predated by common ravens (*Corvus corax*) and Clarion Island whip snakes (*Masticophis anthonyi*). On the other hand, although the environmental conditions are not extremely different between Clarion Island (SST: 26.3°C; CHL 0.09 mg/m^3^, Lerma, Castillo‐Guerrero, et al., 2020), Palmyra Atoll (SST: 25.5–29.9°C; CHL 0.1–0.2 mg/m^3^, Young, Shaffer, et al., 2010), and Tromelin (SST: ~28°C, CHL 0.03–0.13 mg/m^3^, Kappes et al., 2011), the differences in inter‐specific segregation between studies might be related to local‐scale environmental variations. At Palmyra Atoll, red‐footed boobies were suspected to travel to specific areas of the atoll that were slightly more productive (Young, Shaffer, et al., 2010), and in Tromelin, masked boobies use warmer, deeper, and less windy oceanic waters than red‐footed boobies (Kappes et al., 2011). Around Clarion Island, however, remotely sensed environmental data show no major variations within the boobies' foraging range, and thus, segregation between species might be weak. Boobies are known to adjust their foraging strategies to local oceanographic habitats at different locations (Gilmour et al., 2018; Mendez et al., 2017), and thus, the presence or absence of differences in space use between booby species might reflect differences in the local‐scale distribution of their preferred habitats or prey.

### 4.3 Intra‐specific differences

We predicted that there would be intra‐specific differences in the isotopic niches within both booby species, and that these differences would be larger during the breeding than during the non‐breeding period, particularly in the more‐dimorphic species. Our results accordingly showed that female masked boobies had a higher trophic level than males during breeding, but there was no intra‐specific difference during the non‐breeding period or in red‐footed boobies for either period. Sex‐specific differences in masked boobies agreed with results from Rapa Nui, where incubating females had higher δ^15^N values than males, but contrasted with results from Palmyra Atoll, Clipperton Island, Abrolhos, Atol das Rocas, and Fernando de Noronha, where no differences were found (see Table 2). For red‐footed boobies, the lack of isotopic niche differences between females and males agreed with results from Palmyra Atoll and Clipperton Island, but contrasted with those from Europa Island and the Cayman Islands (see Table 2). On Europa Island, chick‐rearing females had similar δ^15^N values but higher δ^13^C values than males (Cherel et al., 2008), and in the Cayman Islands, incubating and chick‐rearing females had higher δ^15^N and δ^13^C values than males (Austin et al., 2021).

The occurrence of intra‐specific differences in one but not the other booby species and the apparent conflicting results of previous studies might be related to four, not mutually exclusive reasons: the degree of sexual dimorphism, their prey distribution, reproductive roles, or the breeding stage studied. First, the degree of sexual size dimorphism in boobies is colony‐specific (Nelson, 1978; Van Oordt et al., 2018), and greater size dimorphism might facilitate competitive exclusion and niche specialization. For example, red‐footed booby females were 5% heavier than males and had a 0.4% larger culmen in the current study (Table 3), whereas females in the Cayman Islands were 15% heavier and had a 3.9% larger culmen than males (Austin et al., 2021) and females at Europa Island were 14% heavier and had a 3.5% larger culmen (Weimerskirch et al., 2006). Second, the occurrence of differences in some but not other booby colonies might due to an effect of environmental conditions on local prey distribution and availability. Foraging behavior in boobies varies according to local oceanographic habitats (Gilmour et al., 2018; Mendez et al., 2017); however, both species in the present study faced similar local oceanographic habitats, suggesting that the differences were likely due to differences in their prey distribution. For example, the environmental conditions in Rapa Nui were homogeneous, and foraging segregation might thus not help to avoid intra‐specific competition for resources (Lerma, Serratosa, et al., 2020). This suggests that the prey of red‐footed boobies at Clarion Island may be distributed more homogeneously than that of masked boobies. Third, the differences may be the result of reproductive role specialization and energetic constraints. Female masked boobies lay two eggs (Lerma, Serratosa, et al., 2020), whereas red‐footed boobies only lay one (Lormee et al., 2005). Female masked boobies might thus have higher nutritional demands (Lerma et al., 2022; Machovsky‐Capuska et al., 2016) and adapt their foraging to compensate for their initial investment in reproduction. Fourth, we studied incubating individuals, but most studies found differences during the chick‐rearing period, particularly for red‐footed boobies. The current study might thus have covered an early part of the breeding period, when competition and the need for resource partitioning is lower compared with the chick‐rearing period, when parents attending a chick are limited to foraging closer to the colony (Lerma, Dehnhard, et al., 2020). Comparative studies considering the degree of sexual size dimorphism, local prey availability, and distribution, and including more breeding stages are needed to test these hypotheses.

### 4.4 Breeding versus non‐breeding period

We predicted that the δ^15^N and δ^13^C values would be similar in the breeding and non‐breeding periods in these booby species because both species are seen at Clarion Island year‐round. Accordingly, red‐footed boobies showed niche overlap and similar δ^13^C values between the breeding and the non‐breeding periods, in agreement with this species being a year‐round resident species at some colonies (Votier et al., 2023). In contrast, masked boobies showed low niche overlap between the breeding and non‐breeding periods and different δ^13^C values. This suggests that masked boobies migrate, in contrast to other studies showing that masked boobies stayed year‐round (Roy et al., 2021); however, we cannot rule out the possibility that masked boobies might perform longer and farther foraging trips during the non‐breeding period, once freed from the constraints of breeding, but still return to the colony to rest. Moreover, the hydro‐geochemical processes responsible for temporal changes in isotope values at the base of the food web are unknown (Espinasse et al., 2022), making it difficult to draw conclusions about the movements of masked boobies and temporal variations in the isoscapes. More information on the non‐breeding movements of these species and a better understanding of the spatio‐temporal changes in the isoscape of the Eastern Tropical Pacific are therefore needed to improve our understanding of the patterns found here.

Despite a lack of information on changes in prey composition and abundances around Clarion Island throughout the year, some flyingfish species form large aggregations during warmer periods of the year, when they mate and spawn close to the surface waters (Ali, 2019; Casazza et al., 2005; Oliveira et al., 2015; Stevens et al., 2003). Aggregation of spawning flyingfish matched our observations of flyingfish containing eggs (4/116 flyingfish prey items of masked boobies). In contrast, squid, as a major prey species of red‐footed boobies, tend to be less seasonal and are available year‐round (Donahue et al., 2020; Granados‐Amores et al., 2010; Harman et al., 1989). We speculate that the breeding period of masked boobies matches a higher availability of their preferred prey, whereas their preferred prey is less available during the non‐breeding period, and thus, masked boobies forage farther away from the colony. In contrast, the breeding period of red‐footed boobies matches when a subset of their prey items becomes more abundant, whereas during the non‐breeding period, red‐footed boobies consume squid or other fish species that are available year‐round in the waters surrounding Clarion Island.

Notably, flyingfish might be particularly important during breeding, as also shown for another marine animal in the Eastern Tropical Pacific, namely, spotted dolphins (*Stenella attenuate*), which prey on a higher proportion of flyingfish than squid during reproduction, attributed to its higher nutritional content compared with squid (Bernard & Hohn, 1989). A higher abundance of more nutritious prey items during breeding would also help to explain why both species showed narrower niche widths during breeding and broader niche widths during the non‐breeding period. Additionally, both species showed a larger niche overlap when their isotopic niche was broader, suggesting that they are more likely to use similar resources when not constrained to central‐place foraging. Furthermore, the presence of subsurface marine predators on Clarion on specific periods of the year may facilitate prey capture for boobies. Boobies form associations with subsurface marine predator species during foraging (Au & Pitman, 1986) and at the Archipelago, Albacore (*Thunnus alalunga*), yellow fin (*T. albacares*), bigeye (*T. obesus*) tuna, and spotted dolphins, which also consume flyingfish (Bernard & Hohn, 1989; Chagnon et al., 2018; Lacerda et al., 2017; Lewallen et al., 2018), are known to occur. Some of these predators are migratory and occur close to the Archipelago only during specific periods of the year (Schaefer et al., 2011). However, further work that investigates the environmental factors determining variations in resource availability and its distribution, as well as the drivers of presence of subsurface marine predators at Clarion Island is necessary. For instance, to determine the influence of the Eastern Pacific Warm Pool and of the California Current on the presence and local‐scale distribution of flyingfish, which in turn attracts subsurface marine predators to the vicinity of Clarion Island.

## 5 CONCLUSIONS

The present study provides new insights into the trophic relationships between masked and red‐footed boobies, which coexist in tropical areas. The continued difference in isotopic niches between the breeding and non‐breeding periods suggests that both species have some degree of niche specialization. We also found evidence of intra‐specific differences, but only during the breeding period, when female masked boobies show higher δ^15^N values than males. In contrast, the foraging ecology of red‐footed boobies seems to be similar in both sexes year‐round. Our results also suggest that red‐footed boobies are more likely to be resident species at Clarion Island, while masked boobies may move farther away; however, more information on the year‐round movements of these species, especially masked boobies, is needed. Overall, these results support the existence of niche partitioning in these taxonomically closely related species, and suggest that intra‐specific niche partitioning is more likely to occur during the breeding than during the non‐breeding period, particularly in the more‐dimorphic species. This study furthers our understanding of the strategies that breeding seabirds use to cope with the same oligotrophic environmental conditions.

## AUTHOR CONTRIBUTIONS

**Miriam Lerma:** Conceptualization (lead); data curation (lead); formal analysis (lead); project administration (lead); visualization (equal); writing – original draft (equal). **Nina Dehnhard:** Conceptualization (equal); writing – review and editing (equal). **José Alfredo Castillo‐Guerrero:** Conceptualization (equal); project administration (equal); writing – review and editing (equal). **Salvador Hernández‐Vázquez:** Project administration (equal); writing – review and editing (equal). **Christian C. Voigt:** Formal analysis (equal); methodology (equal); validation (equal). **Stefan Garthe:** Funding acquisition (lead); writing – review and editing (equal).

## FUNDING INFORMATION

M. Lerma was funded by INAPI‐CONACyT Scholarship no. 411876. Fieldwork was co‐financed by the Research and Technology Centre (FTZ), University of Kiel, and Universidad de Guadalajara.

## CONFLICT OF INTEREST STATEMENT

All authors declare that they have no conflict of interest.

## Data Availability

Data of stable isotopes from red‐footed and masked boobies are archived at Dryad Digital Repository (doi: 10.5061/dryad.573n5tbg4). Functions used for analyses and plotting are available on github (https://github.com/MiriamLL/isoseabird).
